# Complete Genome and Plasmid Sequences of Three Canadian Strains of *Salmonella enterica* subsp. *enterica* Serovar Enteritidis Belonging to Phage Types 8, 13, and 13a

**DOI:** 10.1128/genomeA.01017-15

**Published:** 2015-09-24

**Authors:** Muhammad Attiq Rehman, Geneviève Labbé, Kim Ziebell, Roger P. Johnson, John H. E. Nash

**Affiliations:** a Laboratory for Foodborne Zoonoses, Public Health Agency of Canada, Guelph, Ontario, Canada; b Department of Pathobiology, Ontario Veterinary College, University of Guelph, Guelph, Ontario, Canada

## Abstract

*Salmonella enterica* subsp. *enterica* serovar Enteritidis is a prominent cause of human salmonellosis frequently linked to poultry products. In Canada, *S*. Enteritidis phage types 8, 13, and 13a predominate among both clinical and poultry isolates. Here, we report the complete genome and plasmid sequences of poultry isolates of these three phage types.

## GENOME ANNOUNCEMENT

Nontyphoidal salmonellosis is one of the most common zoonotic foodborne infections worldwide (1). Over the past decade, *Salmonella enterica* subsp. *enterica* serovar Enteritidis has become the predominant serovar isolated from humans in Canada, accounting annually for 30 to 40% of all reported clinical isolates (2–4). Phage typing of *S*. Enteritidis (5) has revealed geographic differences in the predominant phage types (PTs). In Europe, PTs 4, 1, 8, and 21 are the most common (6), whereas in Canada, ~60% of human isolates are PTs 8, 13, and 13a (3, 4). The same three PTs predominate among nonhuman *S*. Enteritidis isolates submitted to our World Organisation for Animal Health (OIE) Reference Laboratory for Salmonellosis, of which ~85% are from chickens or chicken products. Here, we announce the availability of the complete genome and plasmid sequences of one isolate each of PT 8, 13, and 13a from poultry sources in Canada that may serve as reference genomes for comparative analyses.

*S*. Enteritidis poultry isolates H10-082405-11 (PT8), SA01AB08065501 (PT13), and SA01AB10104801 (PT13a) were propagated in tryptic soy broth. Genomic DNA was prepared by using the Qiagen EZ1 DNA tissue kit. Sequencing was performed on two platforms: Pacific Biosciences (PacBio) *RS* II (Menlo Park, CA, USA), and Illumina MiSeq (Illumina, Inc., San Diego, CA). PacBio (performed at the McGill University and Génome Québec Innovation Centre, Montréal, Québec, Canada) generated 326,964 raw subreads of average length 2,885 to 3,240 bp using two single-molecule real-time (SMRT) cells. The contigs had an average coverage of 10.65× to 22.88× and were assembled *de novo* using Hierarchical Genome Assembly Process (HGAP) (7). Sequencing on the Illumina MiSeq platform was performed at the Public Health Agency of Canada (PHAC) National Microbiology Laboratory, Winnipeg, Canada, with 101 × 2 paired-end reads with an average 84.2× coverage after library preparation, using the Illumina Nextera XT kit. The Illumina reads were analyzed and quality checked using FastQC (http://www.bioinformatics.babraham.ac.uk/projects/fastqc/). An *in silico NcoI* restriction map of the GenBank *S*. Enteritidis PT4 strain P125109 (GenBank accession no. AM933172) was generated and used to verify contig assembly using MapSolver version 2.1.1 (OpGen, Inc., Madison, WI). Genome assemblies were created by using the MIRA assembler version 4.9.3 (8) and by manually checking potential joins using the Gap5 software of the Staden package (9). Comparison with the *in silico* restriction map of GenBank strain P125109 and with closely related plasmid sequences in GenBank, together with the finishing process, produced fully assembled genomes and plasmids. The genomes consisted of single chromosomal contigs ranging from 4,679,291 to 4,712,309 bp, with an average G+C content of ~52.17%. The plasmid contigs ranged from 34,541 to 59,372 bp, with G+C content ranging from ~41.45 to 51.96%.

The genomes and plasmids were annotated using the National Center for Biotechnology Information (NCBI) Prokaryotic Genomes Annotation Pipeline (PGAP) (http://ncbi.nlm.nih.gov/genomes/static/Pipeline.html), identifying ~4,447 to 4,486 coding DNA sequences (CDSs) per genome and ~43 to 74 CDSs per plasmid.

### Nucleotide sequence accession numbers.

The complete genome and plasmid sequences of these three *S*. Enteritidis strains have been deposited in GenBank under BioProject no. 219482. The GenBank accession numbers are listed in Table 1.

**TABLE 1:** Accession and isolate numbers for the three *Salmonella* Enteritidis genomes and plasmids sequenced in this study

GenBank accession no.	Isolate accession no.	Original isolate no.	Phage type	PFGE type^a^
CP007329	EC20120002	H10-082405-11	8	SENXAI.0003, SENBNI.0003
KT317612	pSE_EC20120002_60			
CP007249	EC20090641	SA01AB08065501	13	SENXAI.0038, SENBNI.0016
KT317611	pSE_EC20090641_60			
CP007267	EC20120005	SA01AB10104801	13a	SENXAI.0006, SENBNI.0007
KT317613	pSE_EC20120005_60			
KT317614	pSE_EC20120005_35			

a PFGE, pulsed-field gel electrophoresis.
